# LncRNA DRAIC inhibits proliferation and metastasis of gastric cancer cells through interfering with NFRKB deubiquitination mediated by UCHL5

**DOI:** 10.1186/s11658-020-00221-0

**Published:** 2020-04-25

**Authors:** Zheng Zhang, Xiaoxuan Hu, Jia Kuang, Jinmao Liao, Qi Yuan

**Affiliations:** Department of Hepatopathy, Hunan Provincial People’s Hospital, The First Affiliated Hospital of Hunan Normal University, Changsha, 410000 Hunan Province China

**Keywords:** Gastric cancer, Long non-coding RNA DRAIC, Deubiquitination, UCHL5, NFRKB

## Abstract

**Background:**

Long non-coding RNA (lncRNA) as a widespread and pivotal epigenetic molecule participates in the occurrence and progression of malignant tumors. DRAIC, a kind of lncRNA whose coding gene location is on 15q23 chromatin, has been found to be weakly expressed in a variety of malignant tumors and acts as a suppressor, but its characteristics and role in gastric cancer (GC) remain to be elucidated.

**Methods:**

Sixty-seven primary GC tissues and paired paracancerous normal tissues were collected. Bioinformatics is used to predict the interaction molecules of DRAIC. DRAIC and NFRKB were overexpressed or interfered exogenously in GC cells by lentivirus or transient transfection. Quantitative real-time PCR (qPCR) and western blotting were used to evaluate the expression of DRAIC, UCHL5 and NFRKB. The combinations of DRAIC and NFRKB or UCHL5 and NFRKB were verified by RNA-IP and Co-IP assays. Ubiquitination-IP and the treatment of MG132 and CHX were used to detect the ubiquitylation level of NFRKB. The CCK-8 and transwell invasion and migration assays measured the proliferation, migration and invasion of GC cells.

**Results:**

DRAIC is down-regulated in GC tissues and cell lines while its potential interacting molecules UCHL5 and NFRKB are up-regulated, and DRAIC is positively correlated with NFRKB protein instead of mRNA. Lower DRAIC and higher UCHL5 and NFRKB indicated advanced progression of GC patients. DRAIC could increase NFRKB protein significantly instead of NFRKB mRNA and UCHL5, and bind to UCHL5. DRAIC combined with UCHL5 and attenuated binding of UCHL5 and NFRKB, meanwhile promoting the degradation of NFRKB via ubiquitination, and then inhibited the proliferation and metastasis of GC cells, which can be rescued by oeNFRKB.

**Conclusion:**

DRAIC suppresses GC proliferation and metastasis via interfering with the combination of UCHL5 and NFRKB and mediating ubiquitination degradation.

## Introduction

Gastric cancer (GC) is one of the most common malignant tumors of the digestive tract. According to statistics, 1034,000 new cases and 783,000 related deaths resulted from GC in 2018 worldwide. Its incidence ranked sixth among all malignant tumors, but mortality ranked second [1]. The higher mortality rate was due to the serious malignancy of GC cells. In addition, the lack of specific diagnostic markers for early GC and the absence of targeting drugs also contribute to poor prognosis of patients with GC. Therefore, it is crucial to clarify the exact mechanism of the occurrence and development of GC and to develop new targeted drugs and diagnostic kits for improving the prognosis of GC patients.

Long non-coding RNAs (lncRNAs) have become a focus in the field of epigenetics in recent years. They participate in a wide range of biological processes by influencing gene promoter transcription, chromatin remodeling and gene splicing and regulate activity and stability of binding protein [2–6]. Abnormal expression and regulation of lncRNAs were involved in oncogenesis and development, including GC. PVT1, HNRNPKP2, HOXA11-AS and many other lncRNAs can affect the malignant phenotype of GC cells at transcriptional and post-transcriptional levels [7–9]. In 2015, lncRNA Downregulated RNA in cancer (DRAIC) was firstly reported as a target gene of FOXA1 and NKX3–1 to inhibit the ability of transition from an androgen-dependent (AD) to a castration-resistance state, epithelial-mesenchymal transition (EMT) and metastasis of prostate cancer cells, and TCGA database analysis showed that its expression was closely related to the survival rate of patients with malignant tumors such as GC, lung cancer and hepatocellular carcinoma [10, 11]. In addition, high expression of nuclear factor kappa-B (NF-κb) was found in the tumor tissues with inferior DRAIC, and vitro and vivo studies verified that DRAIC could inhibit activation of NF-κb and prostate cancer progressions via binding to subunits of the IκB kinase (IKK) complex [12]. Meanwhile, RNA sequencing (RNA-seq) identified strong down-regulation of DRAIC in retinoblastoma compared with that in normal retina, and restoring DRAIC significantly slowed the growth of the Y79 retinoblastoma cell line [13]. Although the anti-cancer effects of DRAIC have been verified by some studies, there were still several sources of evidence that DRAIC also plays an oncogenic role in other cancers such as breast cancer (BC) and nasopharyngeal carcinoma (NPC). Research showed that DRAIC was highly expressed in BC and NPC, and was positively correlated with pathological stage, lymph node metastasis and poor prognosis of patients [14–16]. Simultaneously, DRAIC could facilitate proliferation of BC independently of autophagy [14] and enhance the malignant phenotype of NPC via adsorbing microRNA-122 and up-regulating special AT rich sequence-binding protein-1 (SATB1). Moreover, a bioinformatics analysis indicated there was abnormal expression of DRAIC in Hirschsprung’s disease, which also suggested the potential value of DRAIC in gastrointestinal cancer [17]. The above studies exhibited dual effects of DRAIC on distinct tumors, but the exact mechanism of its action in GC remains to be clarified.

Ubiquitination is one of the main pathways for the degradation of endogenous proteins, which is an ordered degradation process in which ubiquitin-activated enzymes E1, E2 and E3 were successively linked to degradable proteins and recognized and mediated by proteasome. There are many factors affecting ubiquitination level of proteins, among which deubiquitinase can deubiquitize proteins by hydrolyzing ubiquitin chains and prevent protein degradation through ubiquitination, which is a core factor in the regulation of ubiquitination. Deubiquitinase can be divided into cysteine protease and metalloproteinase. Ubiquitin carboxyl terminal hydrolase (UCHs) belongs to the subclass of cysteine protease, which can hydrolyze polyubiquitin chains into free ubiquitin monomers and reverse ubiquitination modification [18–20]. As one of the isomers of UCHs, UCHL5 participates in the ubiquitination regulation of various proteins and is associated with the occurrence and development of several malignant tumors. However, similar to DRAIC, UCHL5 has been corroborated to play conflicting biological effects in cancers: The superior expression of UCHL5 was observed in esophageal squamous cell carcinoma tissues, and was correlated with TNM stage, lymph node metastasis, recurrence rate and poor prognosis [21]. Also, bis-benzylidene piperidone RA190 could restrict mice xenografts’ growth of colorectal cancer via inactivating UCHL5 [22]. Small molecule inhibitors of UCHL5 could induce apoptosis of multiple myeloma cells and reverse bortezomib resistance [23]. In addition, positive cytoplasmic UCHL5 indicated that patients with GC had smaller primary lesions and TNM stage I-II, and predicted longer survival time [24], while positive nuclear UCHL5 implied a better prognosis of patients aged over 65 years or with lymph-node positivity [25]; at the same time, high and undetectable UCHL5 levels were related to extended overall disease-specific survival in the lymph-node positivity rectal cancer patients [26]. In this study, primary bioinformatics analysis shows that DRAIC has a potential for binding with UCHL5, which has aroused our interest.

Nuclear factor related to κB binding protein (NFRKB or INO80G) is a pivotal chromatin remodeling molecule. As a target molecule of UCHL5, NFRKB is protected from ubiquitination degradation by UCHL5 and plays an important role in DNA double-strand break (DSB) resection and repair by homologous recombination, and is regarded as a potential therapeutic target for cancer [27, 28], but the mechanism remains to be investigated. As bioinformatics evidence, we further analyzed the expression characteristics and correlation of DRAIC, UCHL5 and NFRKB in GC tissues and cell lines, whose results showed the down-regulation of DRAIC and up-regulation of UCHL5 and NFRKB in GC. Further studies showed that DRAIC can promote the ubiquitination degradation of NFRKB mediated by UCHL5, and confirmed the inhibitory effect of DRAIC on proliferation and metastasis of GC cells, which could be rescued by oeNFRKB. Our study suggests a novel ubiquitinated regulatory model of UCHL5/NFRKB in GC mediated by lncRNA DRAIC.

## Materials and methods

### Clinical tissue specimens

Sixty-seven pairs of GC tissues and corresponding paracancerous normal control (NC) tissues were collected from 67 GC patients who underwent surgical resection for primary GC from January to December 2018 in Hunan Provincial People’s Hospital. None of the patients had received any form of antineoplastic therapy prior to surgical resection. Tissues were snap-frozen and stored in liquid nitrogen for the extraction of RNA. All patients provided written informed consent for the use of the tumor tissues for clinical research.

### Cell culture and treatments

The cells used in this study included: human embryonic kidney cell 293 T, human GC cell lines HGC-27, SGC-7901, BGC-823, AGS and MKN45, and human gastric epithelial cell GES-1. All of the cell lines are ATCC (American Type Culture Collection) sources and were purchased from the Chinese Academy of Sciences (Shanghai, China). 293 T were cultured in DMEM high glucose culture fluid (Gibco Company, USA) with 10% Fetal Bovine Serum (FBS, HyClone, Australia) and 1% penicillin-streptomycin (Gibco Company, USA). HGC-27, SGC-7901, BGC-823, AGS, MKN45 and GES-1 were cultured in RPMI-1640 Medium (Thermo Scientific, USA) with 10%FBS and 1% penicillin-streptomycin. Supportive culture environment: 37 °C, 5% CO_2_ with 100% relative humidity. Treatment with a gradient concentration of cycloheximide (CHX, 5 μM, 0, 6 and 12 h) and proteasome inhibitor MG132 (0 and 5 μM, 12 h) in cell lines was used to observe the effect of DRAIC on the ubiquitination level of NFRKB.

### Lentivirus transfection and transient transfection

Lentivirus transfection: 293 T cells were cultured in 6-well plates with 80% cell density. 0.75 μg Gag-pol expression vector, 0.30 μg Rev. expression vector and 0.45 μg VSV-G expression vector were diluted in 1.5 mL of DMEM separately with 1.5 μg of target plasmid to be packaged with plvx-DRAIC-Puro (oeDRAIC), Plvx-puro1 (Vector1) (Vigenebio, China) and GV248 (shControl), shDRAIC#1 and shDRAIC#2 (Genechem, China). 5 min later, 7.5 μL of Lipofectamine 2000 transfection reagent (Invitrogen, USA) was fully mixed with plasmids. 293 T cells were incubated with a mixture containing plasmids for 6 h, then cultured with 10%FBS + DMEM for 48 h. Cell supernatant regarded as a viral suspension was collected and filtered with a 0.45 μm filter (Millipore, USA). HGC-27 and MKN45 or SGC-7901 were cultured by the viral suspension mixed with 10%FBS + RPMI-1640 medium in the ratio of 1:1 for 3 days adding 1/1000 polybrene (Sigma-Aldrich, USA). Puromycin (Thermo Scientific, USA) screened cells until the steady growth state.

Transient transfection: 1.5 μg plvx-NFRKB-Puro (oeNFRKB) or Plvx-puro2 (Vector2) (Vigenebio, China) was mixed with 7.5 μL of Lipofectamine 2000 diluted in 1.5 mL of RPMI-1640 medium for 20 min. Cells were cultured with the mixture for 6 h, and then cultured with 10%FBS + RPMI-1640 medium for 48 h.

### Quantitative real-time PCR (qPCR)

Total RNA of cells or tissues was extracted by TRIzol (Invitrogen, USA), and the quality of total RNA was detected at an A260/A280 ratio using quantification by NanoDrop (Thermo Scientific, USA). 1 μg of RNA was reverse-transcribed to cDNA using the PrimeScript Strand cDNA Synthesis Kit (Takara, Japan). qPCR analysis of lncRNA DRAIC, UCHL5 and NFRKB was performed on an Applied Biosystems ABI Prism 7500 sequence detection system, and RT products were quantified with SYBR Green real-time PCR (Takara, Japan). The expression of DRAIC, UCHL5 and NFRKB was normalized to that of β-Actin using the 2^−ΔΔCt^ method. The sequences of β-Actin primers were: 5′-TGGCACCCAGCACAATGAA-3′ (forward); 5′-CTAAGTCATAGTCCGCCTAGAA-3′ (reverse). The sequences of DRAIC primers were: 5′-GTCTCAAACTCCCGACCTCA-3′ (forward); 5′-CAACCAGCTTGTGAGGCATT-3′ (reverse). The sequences of UCHL5 primers were: 5′-TTCGATGTCTCTAGGGTGGC-3′ (forward); 5′-GATCCACCTCTCGCTCTCAG-3′ (reverse). The sequences of NFRKB primers were: 5′-TGAAGACAGCTCAGATGCCA-3′ (forward); 5′-CTTGTCAAACACGCCCTTCA-3′ (reverse).

### Western blotting (immunoblotting, IB)

0.5 ml, 4 °C Cell lysis buffer IP (Beyotime, China) containing protease and phosphatase inhibitor cocktail was used to lyse cells (3 × 10^5^) and tissues (1 mg, grinded) at 0 °C for 2 h to extract total protein. We applied centrifugation at 4 °C, 15000 r/min, collected the supernatant, trimmed the protein system and denatured at 95 °C for 5 min. Proteins were separated by 10% SDS-PAGE electrophoresis and transferred to PVDF (0.45 μm, Thermo Scientific) membrane. 5% skim milk was used to block membrane for 2 h. The relevant first antibodies were used to incubate PVDF membrane at 4 °C overnight; information on antibodies is as follows: anti-UCHL5 (1:1000 diluted, 38KDa, Abcam, ab133508); anti-NFRKB (1:500 diluted, 139KDa, Abcam, ab86154); anti-Ubiquitin (1:2000 diluted, Abcam, ab7780); anti-β-Actin (1:2000 diluted, 42 kDa, abcam, ab8226). The relevant second rabbit anti-mouse or anti-rabbit antibody (1:2000 diluted, abcam, ab6728 or ab6721) incubated strip at room temperature for 1 h. ECL detection reagent and Bio-Rad GelDoc XR were used to detect the intensity of the strip signal.

### RNA immunoprecipitation (RIP)

RIP: Cells were lysed by cell lysis buffer IP. 1.5 mL of RIP buffer (Promega, USA) resuspend the cell lysate which was divided into 3 fractions including Input, anti-IgG and anti-UCHL5. 2 μg of anti-IgG (ab131368) or anti-UCHL5 antibody (ab133508) incubated the cell lysate at 4 °C for 12 h with gentle rotation. 40 μL of protein A/G beads (Santa Cruz) were added into lysate at 4 °C for 12 h with gentle rotation. The supernatant was removed after the beads were pelleted at 2500 rpm for 30 s. The beads were washed with RIP buffer 3 times. Extraction of total RNA by TRIzol for qPCR was performed.

### Co-Immunoprecipitation (co-IP) and ubiquitination-IP (Ubi-IP)

Co-IP: Cells pre-treated with 2 μg of oeNFRKB plasmid for 48 h (In order to maintain the same amount of NFRKB). Cells were rinsed with ice-cold PBS and solubilized for 15 min on ice in cell lysis buffer IP supplemented with protease inhibitors. Lysates were then centrifuged in a microfuge at 12,000 rpm for 10 min, and the supernatants were immunoprecipitated at 4 °C with UCHL5 (Co-IP) or NFRKB (Ubi-IP) antibodies (5 mg/ml) for 4 h followed by protein A/G beads overnight. After centrifugation, beads were washed five times with RIPA buffer. Bound proteins were eluted with 2X SDS sample buffer and subjected to SDS-PAGE. Proteins resolved on SDS-PAGE were transferred to PVDF membranes. Blots were incubated at room temperature for 1 h in 5% skim milk, followed by incubation with indicated antibodies at 4 °C overnight. The following steps are the same as western blotting.

### CCK-8 assay

Cells were seed into 96-well plates at 1 × 10^4^ cells/well overnight. CCK-8 reagent (Dojindo, Japan) mixed with 10%FBS + RPMI-1640 medium in a 1:9 ratio was used to culture cells for 3 h avoiding light. Absorbance at 450 nm at 0, 24, 48 and 72 h was measured by the multi-plate reader (Bio-Rad, USA). Cell proliferation abilities were demonstrated by the ratio of absorbance at 24, 48 and 72 h to that at 0 h. Cell proliferation was determined by 5 independent experiments.

### Transwell invasion and migration assay

Transwell invasion assay: Matrigel basement membrane matrix (Corning, USA) was diluted to 50 mg/L to coat the transwell chamber (Corning, USA) at 37 °C for 6 h. 1 × 10^5^ cells resuspended by serum-free RPMI-1640 medium were added to the upper chamber after solidification of Matrigel matrix, and 600 μL of 10%FBS + RPMI-1640 medium was added to the lower chamber. Cells were incubated in humidified air at 37 °C with 5% CO_2_ for 24 h, and then fixed with absolute ethanol for 10 min. 0.1% crystal violet was used to dye cells in the lower chamber for 20 min. There were 5 fields in each well under a 40 × 10 microscope (DM1000, Leica, Germany) randomly, and ImageJ software (National Institutes of Health, USA) was used for cell counting.

Transwell migration assay: a non-Matrigel matrix chamber was substituted for the invasion chamber, and cell processing was the same as before.

### Statistical analysis

All statistical analyses were performed using the IBM SPSS Statistics v17 software package. The quantitative data were compared and analyzed with Student’s t test and analysis of variance (ANOVA). The chi-square test was used to analyze qualitative data. Pearson’s correlation was used for correlation analysis. A two-tailed value of *P* < 0.05 was considered statistically significant.

## Results

### The expression relationships and clinicopathological values of DRAIC, UCHL5 and NFRKB in the GC patient tissues

**Fig. 1:**
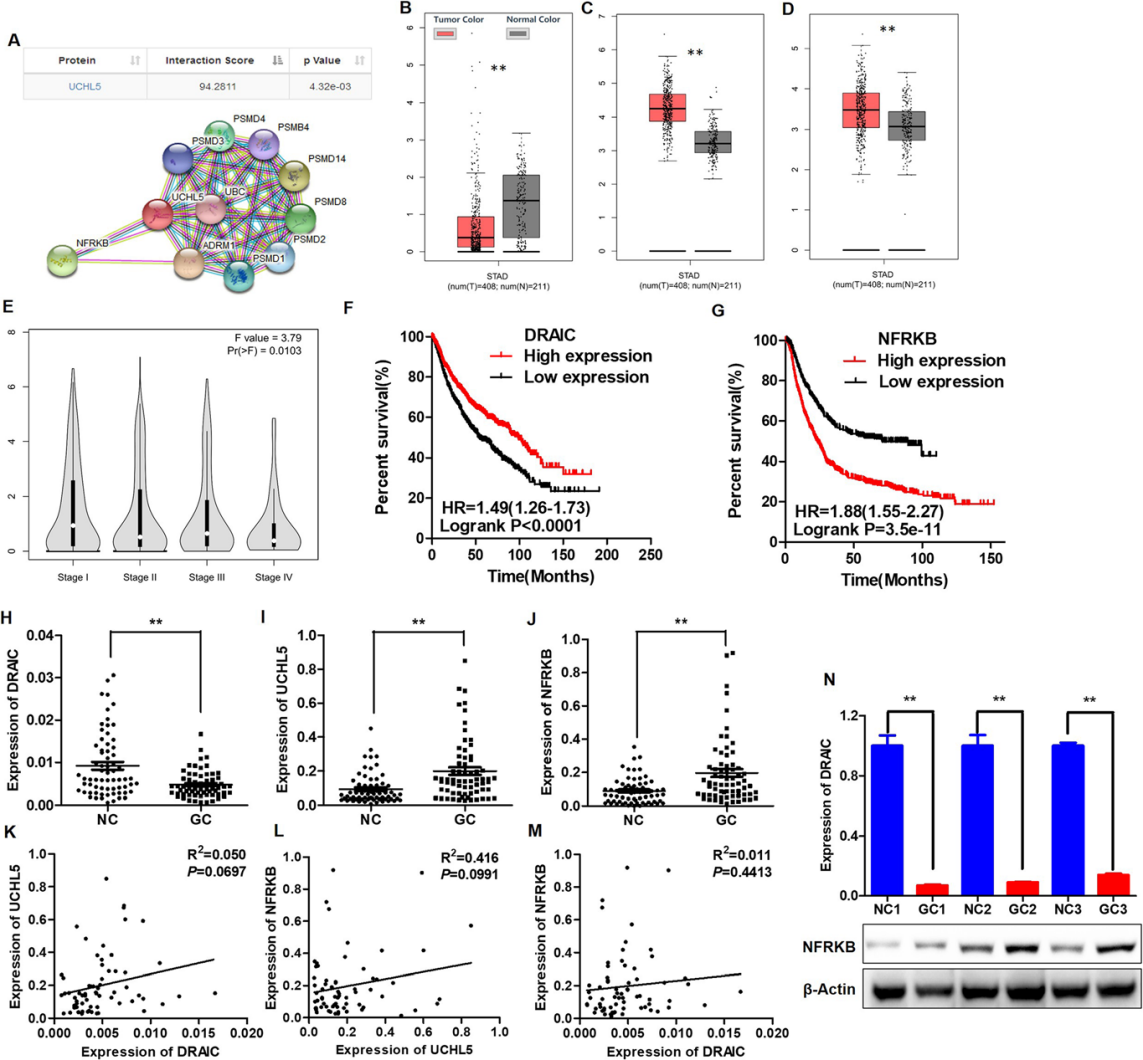
Expression and correlations of DRAIC, UCHL5 and NFRKB in GC tissues. **a.** Bioinformatics analysis of interaction molecules of DRAIC. **b.** DRAIC expression in 408 cases of GC tissues and 211 cases of NC tissues from TCGA database. **c.** UCHL5 expression in 408 cases of GC tissues and 211 cases of NC tissues from TCGA database. **d.** NFRKB expression in 408 cases of GC tissues and 211 cases of NC tissues from TCGA database. **e.** DRAIC respective expression in GC patients with TNM stage I-IV. **f.** Kaplan-Meier curves for overall survival analysis as it correlates to DRAIC expression. **g.** Kaplan-Meier curves for overall survival analysis as it correlates to NFRKB expression. **h.** DRAIC expression in 67 paired GC tissues and NC tissues was determined by qPCR. **i.** UCHL5 expression in 67 paired tissues **j.** NFRKB expression in 67 paired tissues **k.** Analysis of the correlation of DRAIC and UCHL5 mRNA by Pearson correlation coefficient. **l.** Analysis of the correlation of UCHL5 mRNA and NFRKB mRNA by Pearson correlation coefficient. **m.** Analysis of the correlation of DRAIC and NFRKB mRNA by Pearson correlation coefficient. **n.** Analysis of the relationship between DRAIC and NFRKB protein. R^2^ = coefficient of determination. Quantitative data are presented as means ± SD. **P < 0.01 compared with the NC tissues

Considering the potential research value of DRAIC in gastric cancer, we analyzed its interaction molecules and found that there was a potential interaction between DRAIC and UCHL5 (Fig. 1a top, https://annolnc.cbi.pku.edu.cn), and NFRKB, the downstream gene of UCHL5, is one of the potential targets of malignant tumors [28] (Fig. 1a bottom, https://string-db.org/), which naturally attracted our attention. Therefore, we analyzed the expression of DRAIC, UCHL5 and NFRKB in GC through the TCGA database (gepia.cancer-pku.cn/index.html), and found that DRAIC was down-regulated in GC tissues, while both UCHL5 and NFRKB were highly expressed (Fig. 1b, c and d), which suggested that they may participate in the formation of GC. Furthermore, we observed that the expression of DRAIC decreased with the progression of gastric cancer (Fig. 1e), while the low expression of DRAIC and high expression of NFRKB were extremely adverse for the prognosis of GC patients (Fig. 1f and g). The above bioinformatics results suggested that DRAIC, UCHL5 and NFRKB may interact with each other and exert biological effects in GC. To verify the above inference, we further found the low expression state of DRAIC (Fig. 1h) and the high expression state of UCHL5 and NFRKB mRNA (Fig. 1i, j) in 67 pairs of GC and its corresponding paracancerous NC tissues. The correlation analysis shows the expression of DRAIC, UCHL5 and NFRKB mRNA, but no significant correlations were observed (Fig. 1k, l and m), which suggested that the regulation of DRAIC on NFRKB is at the protein level, just like UCHL5 [28]. Then we selected three clinical tissue samples with the lowest expression of DRAIC for Western blotting detection, and found that DRAIC and NFRKB protein expression showed a certain correlation (Fig. 1n). The results of the chi-square test showed that the low expression of DRAIC and the high expression of UCHL5 were related to the lymph node metastasis rate of GC patients (Tables 1, 2), while the high expression of NFRKB indicated the higher TNM stage and lymph node metastasis rate (Table 3).

**Table 1:** Association of DRAIC with clinicopathological variables in GC patients

Index	DRAIC		χ^2^	*P*
	Low	High		
Age, years				
≤ 65	18	15	0.729	0.393
> 65	15	19		
Gender				
Male	14	17	0.387	0.543
Female	19	17		
TNM stage				
I-II	9	16	2.803	0.094
III-IV	24	18		
T-				
T1-T2	11	18	2.623	0.105
T3-T4	22	16		
N-				
pN0	6	16	6.332	0.019
pN+	27	18		
M-				
pM0	21	27	2.051	0.152
pM+	12	7		
Laurén				
Intestinal	10	14	0.861	0.353
Diffuse	23	20

**Table 2:** Association of UCHL5 with clinicopathological variables in GC patients

Index	UCHL5		χ^2^	*P*
	Low	High		
Age, years				
≤ 65	14	19	1.214	0.271
> 65	19	15		
Gender				
Male	18	13	1.792	0.181
Female	15	21		
TNM stage				
I-II	15	10	1.843	0.175
III-IV	18	24		
T-				
T1-T2	18	11	3.360	0.067
T3-T4	15	23		
N-				
pN0	16	6	7.221	0.007
pN+	17	28		
M-				
pM0	26	22	1.635	0.201
pM+	7	12		
Laurén				
Intestinal	15	9	2.625	0.105
Diffuse	18	25

**Table 3:** Association of NFRKB with clinicopathological variables in GC patients

Index	NFRKB		χ^2^	*P*
	Low	High		
Age, years				
≤ 65	13	20	2.529	0.112
> 65	20	14		
Gender				
Male	12	19	2.566	0.109
Female	21	15		
TNM stage				
I-II	17	8	5.607	0.018
III-IV	16	26		
T-				
T1-T2	17	12	1.795	0.180
T3-T4	16	22		
N-				
pN0	16	6	7.221	0.007
pN+	17	28		
M-				
pM0	27	21	3.315	0.069
pM+	6	13		
Laurén				
Intestinal	14	10	1.233	0.267
Diffuse	19	24		

### DRAIC and NFRKB expression in GC cell lines and the effect of DRAIC on UCHL5 and NFRKB

**Fig. 2:**
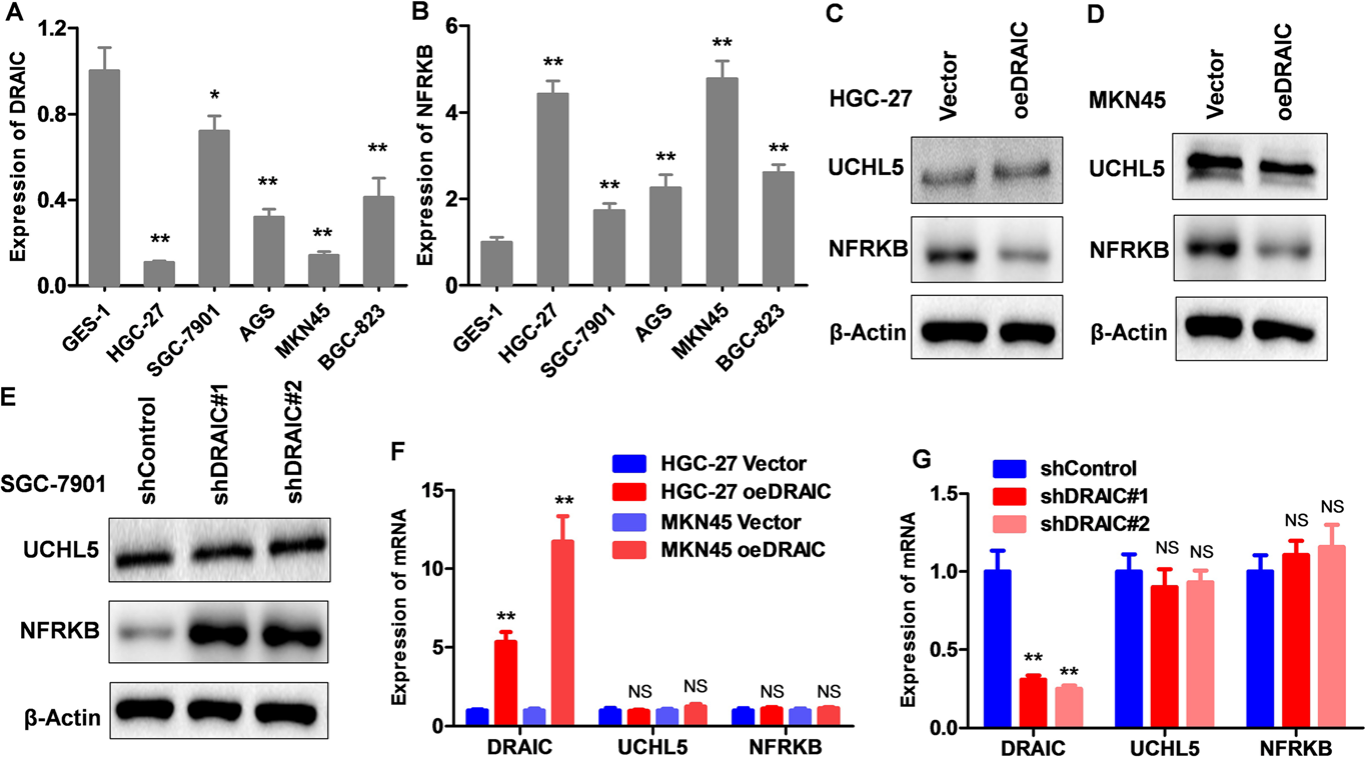
Construction of oeDRAIC and shDRAIC cell lines and regulation of DRAIC on UCHL5 and NFRKB. **a.** Expression of DRAIC in GC and gastric mucosal epithelial cell lines. **b.** Expression of NFRKB in GC and gastric mucosal epithelial cell lines. **c.** The effect of oeDRAIC on UCHL5 and NFRKB protein in HGC-27. **d.** The effect of oeDRAIC on UCHL5 and NFRKB protein in MKN45. **e.** The effect of shDRAIC on UCHL5 and NFRKB protein in SGC-7901. **f.** The effect of oeDRAIC on UCHL5 and NFRKB mRNA in HGC-27 and MKN45. **g.** The effect of shDRAIC on UCHL5 and NFRKB mRNA in SGC-7901. Quantitative data are presented as means ± SD. NS = not significant, *P < 0.05, **P < 0.01 compared with the control group

Based on the significant differential expression of DRAIC in GC tissue and its possible regulatory effect on NFRKB, we detected the expression levels of DRAIC and NFRKB in GC cell lines, and found that the low expression of DRAIC and the high expression of NFRKB in GC cells were comparable with those in gastric mucosal epithelial cells (Fig. 2a, b), which not only clarified the expression characteristics of DRAIC and NFRKB in GC cell lines, but also provided a basis for the construction of cell lines. We overexpressed DRAIC in HGC-27 and MKN45 cells which express relatively lower DRAIC in GC cell lines, and knocked down DRAIC in SGC-7901 cells expressing relatively high DRAIC. We found that oeDRAIC could significantly inhibit NFRKB protein in HGC-27 and MKN45 cells (Fig. 2c, d), but had no significant effect on UCHL5 and NFRKB mRNA (Fig. 2c, d, f). The expression of NFRKB protein in SGC-7901 cells was significantly up-regulated after DRAIC knockdown, but there was similarly no significant change in UCHL5 or NFRKB mRNA expression (Fig. 2e, g), which suggested that DRAIC has a significant negative regulatory effect on NFRKB, and plays this role at the post-translation level without affecting the expression of its potential binding protein UCHL5. Combined with the above clues provided by bioinformatics, we speculated that DRAIC might interfere with the ubiquitination of NFRKB by combining with UCHL5, and then mediate the ubiquitination degradation of NFRKB at the protein level.

### The effect of DRAIC on the combination of UCHL5 and NFRKB

**Fig. 3:**
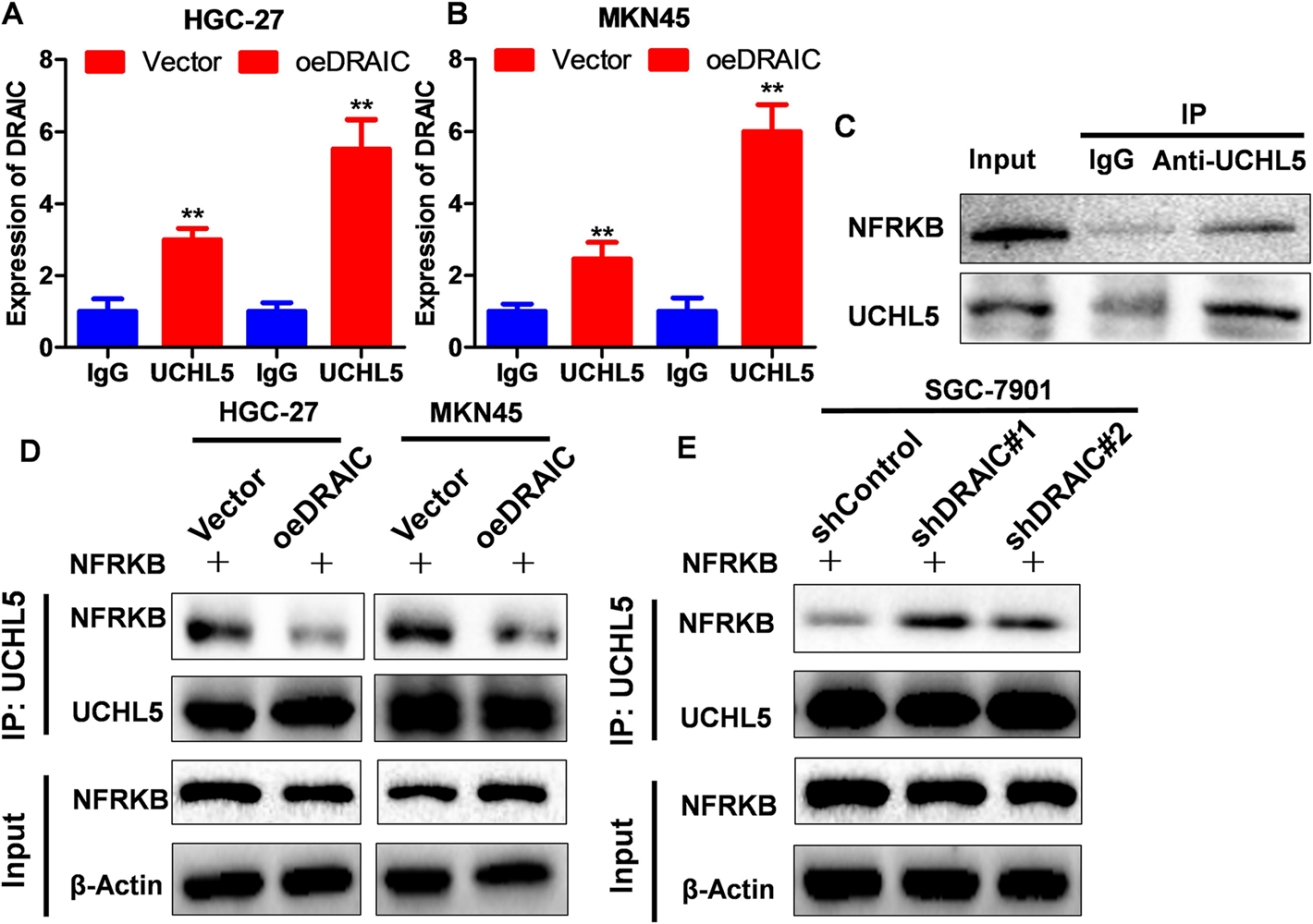
The combinations of DRAIC and UCHL5 and the effects of DRAIC on the binding of NFRKB and UCHL5. **a.** The combination of DRAIC and UCHL5 in HGC-27 Vector and oeDRAIC cells. **b.** The combination of DRAIC and UCHL5 in MKN45 Vector and oeDRAIC cells. **c.** The combination of UCHL5 and NFRKB in HGC-27 cell. **d.** The combination strength of UCHL5 and NFRKB in HGC-27/MKN45 Vector and oeDRAIC cells. **e.** The combination strength of UCHL5 and NFRKB in SGC-7901 shControl and shDRAIC cells. Quantitative data are presented as means ± SD. **P < 0.01 compared with the IgG group

Through prediction of bioinformatics, we found that there is a potential combination of UCHL5 and DRAIC (Fig. 1.A top). Furthermore, we confirmed this combination via RNA-IP assay (Fig. 3a, Fig. 3b). Similarly, the results of bioinformatics analysis (Fig. 1a bottom) and Co-IP assay (Fig. 3c) demonstrated that UCHL5 interacted with NFRKB, which is consistent with the research by *Sahtoe DD* et al. [27]. However, the effect of DRAIC on the combination of UCHL5 and NFRKB was still unclarified, so we detected this combination in oeDRAIC and shDRAIC cell lines, whose results showed that oeDRAIC can significantly reduce the level of NFRKB coprecipitated by UCHL5 (Fig. 3d), while shDRAIC can increase NFRKB binding with UCHL5 (Fig. 3e), which confirmed the speculation that DRAIC may indirectly down-regulate the expression of NFRKB through affecting the deubiquitination induced by UCHL5.

### The effect of DRAIC on the ubiquitylation degradation of NFRKB

**Fig. 4:**
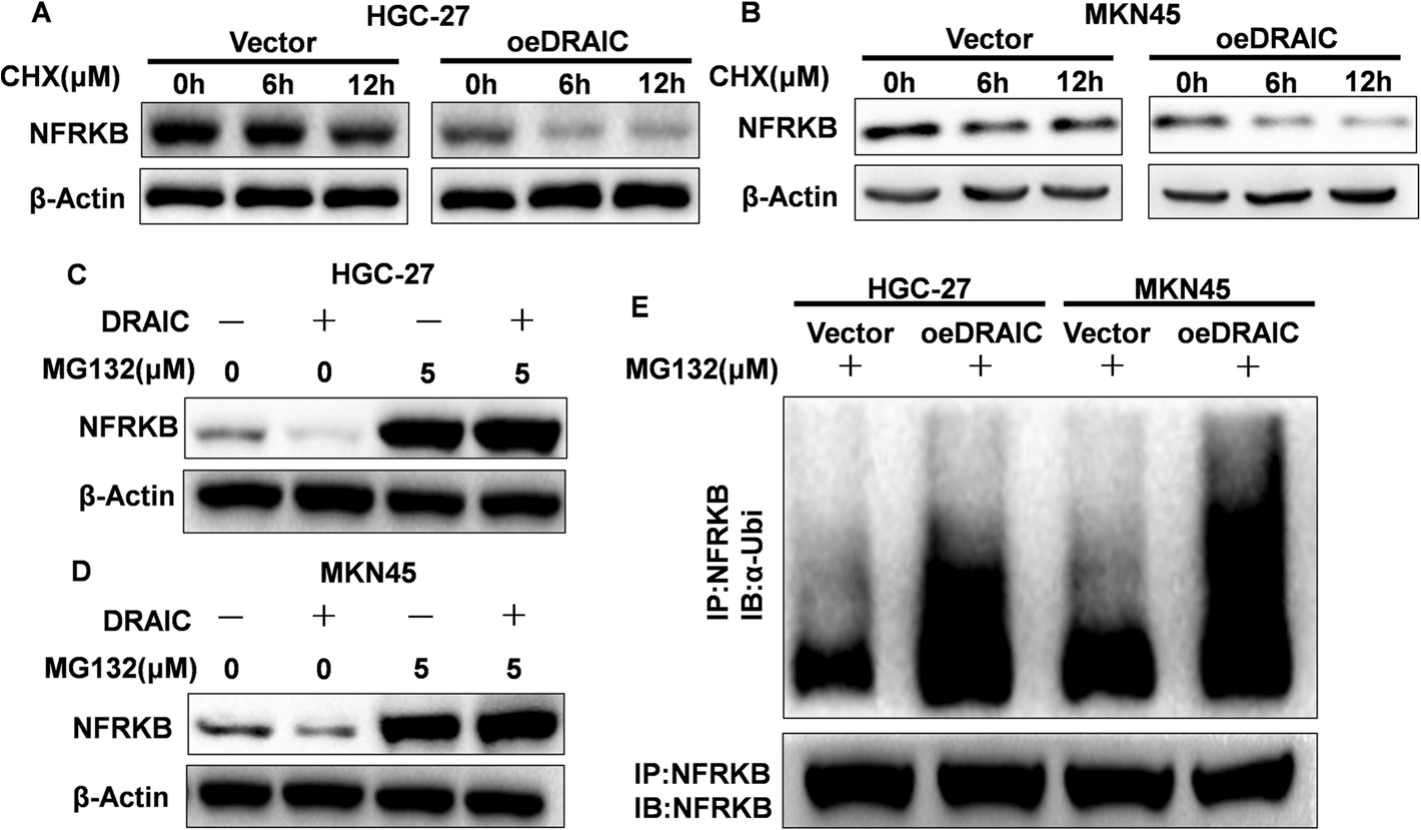
The effect of DRAIC on the degradation and ubiquitination of NFRKB. **a.** The effect of oeDRAIC on the degradation of NFRKB in HGC-27. **b.** The effect of oeDRAIC on the degradation of NFRKB in MKN45. **c.** The blocking effect of proteasome inhibitor on the degradation of NFRKB mediated by oeDRAIC in HGC-27. **d.** The blocking effect of proteasome inhibitor on the degradation of NFRKB mediated by oeDRAIC in MKN45. **e.** The effect of oeDRAIC on the ubiquitination level of NFRKB

To verify the above speculation, we treated Vector and oeDRAIC cells with CHX and observed the degradation rate of NFRKB, and the results demonstrated that the degradation rate of NFRKB in oeDRAIC cells was dramatically accelerated compared with Vector cells (Fig. 4a, b). At the same time, MG132 could block the down-regulation of DRAIC on NFRKB (Fig. 4c, d), which indicated that DRAIC could promote the degradation rate of NFRKB mediated by ubiquitination. So we further tested the ubiquitination level of NFRKB, and found that the NFRKB ubiquitination level increased significantly after oeDRAIC (Fig. 4e), which could demonstrate that DRAIC weakens the deubiquitination of NFRKB mediated by UCHL5, and maintains the ubiquitination level of NFRKB and boost the degradation of NFRKB via the ubiquitination-proteasome pathway.

### Biological effects of oeDRAIC and rescued by oeNFRKB on GC cells

**Fig. 5:**
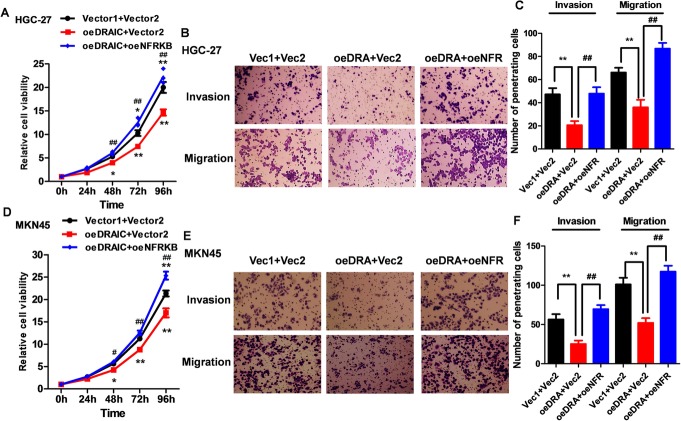
Biological effects of oeDRAIC and oeNFRKB on GC cells. **a.** The effects of oeDRAIC and NFRKB rescued on HGC-27 cell proliferation. **b.** The effects of oeDRAIC and NFRKB rescued on HGC-27 cell metastasis. **c.** Quantitative results of ImageJ analysis Fig. 5b. **d.** The effects of oeDRAIC and NFRKB rescued on MKN45 cell proliferation. **e.** The effects of oeDRAIC and NFRKB rescued on MKN45 cell metastasis. **f.** Quantitative results of ImageJ analysis Fig. 5e. Quantitative data are presented as means ± SD. *P < 0.05, **P < 0.01 compared with the vector1 + vector2 group, ^##^*P* < 0.01 compared with the oeDRAIC+vector2 group

Based on the differential expression of DRAIC in GC and NC tissues and its potential role in several malignant tumors [10–17], we decided to explore the relationship between DRAIC/NFRKB and malignant phenotype of GC cells. It was found that DRAIC can restrain the proliferation abilities of HGC-27 and MKN45 cells (Fig. 5a, d), while the results of transwell invasion and migration assay demonstrated that DRAIC could weaken the metastatic abilities of HGC-27 and MKN45 cells (Fig. 5b, c and Fig. 5e, f). Simultaneously, in order to reveal the biological effects of NFRKB inhibited by DRAIC on GC, we overexpressed NFRKB to rescue the expression of NFRKB exogenously in oeDRAIC cell lines. It was found that oeNFRKB could alleviate the anti-cancer effect induced by DRAIC in HGC-27 and MKN45 cells, suggesting that DRAIC plays an anti-cancer role by suppressing NFRKB.

### Biological effects of shDRAIC on GC cells

**Fig. 6:**
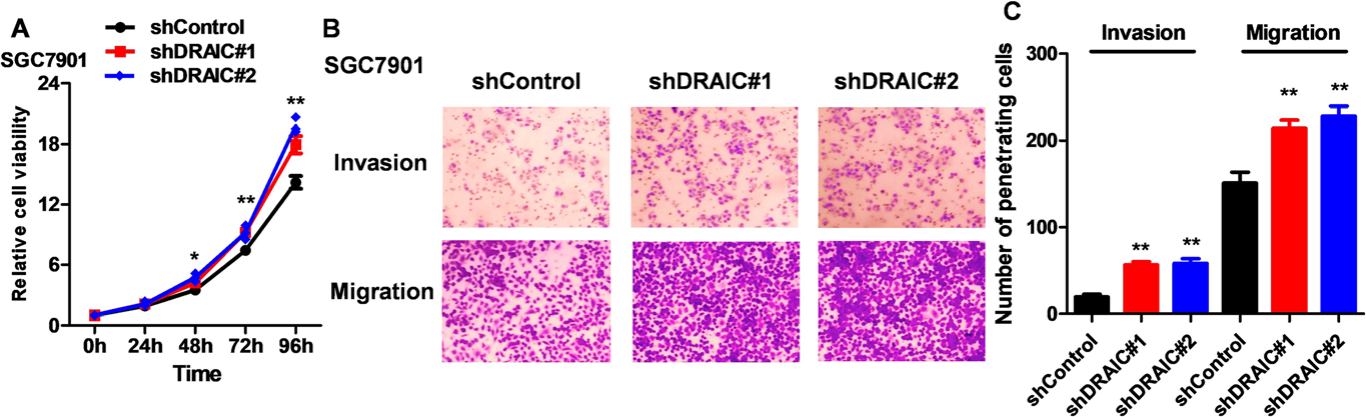
Biological effects of shDRAIC on GC cells. **a.** The effects of shDRAIC on SGC-7901 cell proliferation. **b.** The effects of shDRAIC on SGC-7901 cell metastasis. **c.** Quantitative results of ImageJ analysis Fig. 6b. Quantitative data are presented as means ± SD. *P < 0.05, **P < 0.01 compared with the shControl group

Further, the verification that biological effects of shDRAIC is very necessary for supporting DRAIC as a tumor suppressor on GC. After knockdown of the expression of DRAIC, the proliferation of SGC-7901 increased significantly (Fig. 6a) and more invasive and migrating cells were observed (Fig. 6b, c), which confirmed that DRAIC can inhibit the malignant behavior of GC cells from a negative perspective.

## Discussion

Epigenetic regulation, including transcriptional and post-transcriptional regulation, plays a crucial role in malignant tumors. Ubiquitination, as a critical component of post-translational modification, is the main path of protein degradation and participates in the disorder of oncogenes and anti-oncogenes in tumors. The balance between ubiquitination and deubiquitination is determined by ubiquitin-related enzymes, such as UCHs. Therefore, many UCHs, including UCHL1, UCHL3 and UCHL5, have been found to be associated with the occurrence and development of malignant tumors via regulating the ubiquitin of target genes: oncogene Akt2 and hypoxia inducible factor 1α (HIF-1α) could be maintained by UCHL1-mediated deubiquitination and then promote growth of cancer cells [29–31]. UCHL3 enhanced the DNA damage repair mediated by radiotherapy and chemotherapy in cancer cells via binding to tyrosyl DNA phosphodiesterase 1 (TDP1), a chromosome breakage repair related enzyme, and mediating TDP1 deubiquitination [32–34]. UCHL5 could stabilize the seven-pass transmembrane receptor Smoothened (Smo) protein via deubiquitination and further activated the Hedgehog (Hh) signaling pathway to maintain tissue homeostasis of cancers [35]. Beyond that, dephosphorylation of Smad2 could also be mediated by UCHL5, which plays a crucial role in suppressing cell apoptosis and sustaining cell survival [36]. Although positive cytoplasmic UCHL5 was regarded as a protective marker in GC [24], which seems to be inconsistent with the results of this study, we believe that this inconsistency is related to the difference in the distribution of UCHL5 in the cytoplasm and nucleus. Due to the extensive deubiquitination of UCHL5, the distribution of UCHL5 determines its downstream target protein, and therefore exerts different biological effects. We will also focus on the influence of the UCHL5 distribution on GC cells in further research. This evidence suggested that UCHL5 plays a critical role in a variety of tumors.

LncRNAs are vital molecules in epigenetic regulation of organisms, involving post-translational modifications of various proteins, including phosphorylation [5, 37] and acetylation [38, 39]. Certainly, a notable proportion of lncRNAs have also been found to regulate protein expression through ubiquitination: lncRNA uc.134 could repress hepatocellular carcinoma progression by inhibiting the CUL4A-mediated ubiquitination of LATS1 and increasing YAPS127 phosphorylation [40]. LncRNA ANCR could increase the intensity of phosphorylation at Thr-345 and Thr-487 sites of EZH2 to facilitate EZH2 ubiquitination and hence its degradation [41]. LncRNA UPAT interacts with and stabilizes the epigenetic factor UHRF1 by interfering with its β-transducin repeat-containing protein (TrCP)-mediated ubiquitination [42]. HULC upregulated ubiquitin-specific peptidase 22 (USP22), leading to the decrease of ubiquitin-mediated degradation of Sirt1 protein by removing the conjugated polyubiquitin chains from Sirt1 [43]. Numerous studies, including the above reports, have established the important role of lncRNAs in post-translational modification of tumors, especially ubiquitination. However, a large amount of work is still needed to complete the exact blueprint of the regulatory network.

The lncRNA DRAIC gene, which is located on human chromosome 15q23, was reported as a novel tumor regulator in several cancers recently, but it seemed to exhibit different biological effects in cancer cells from different sources [10–17]. On account of the potential value of DRAIC as a GC marker and target, we intended to reveal the expression characteristics and precise mechanism of DRAIC. Firstly, lower expression of DRAIC was found in GC tissues and cell lines, and indicated the higher lymph node metastasis rate of GC patients, which indicated that DRAIC may play a role as a potential tumor suppressor gene in GC. So we predicted potential interacting molecules of DRAIC by bioinformatics analysis, and the results revealed that UCHL5 may interact with DRAIC as a binding protein. Considering the potential value of UCHL5/NFRKB in cancer [27, 28], we analyzed the expression of UCHL5 and NFRKB in GC through the TCGA database and qPCR, whose results demonstrated that UCHL5 and NFRKB were up-regulated in GC. Meanwhile, the higher expression levels of UCHL5 and NFRKB implied advanced progression, and the higher expression of NFRKB was closely related to the poor prognosis of GC patients. In addition, there was a certain negative correlation between DRAIC and NFRKB protein expression, but a correlation with mRNA level was not observed, which suggested that DRAIC regulates NFRKB at the post-transcriptional level. Therefore, we overexpressed or interfered with DRAIC in GC cell lines with different DRAIC expression backgrounds exogenously and detect the mRNA and protein expression of UCHL5 and NFRKB, and the results revealed the negative regulation of DRAIC on NFRKB protein, instead of UCHL5 or NFRKB mRNA. Although the predicted binding protein of DRAIC was UCHL5, it only regulated NFRKB protein as a downstream gene of UCHL5. Considering the deubiquitination function of UCHL5, we speculated that DRAIC might regulate the ubiquitination level of NFRKB by influencing the binding of UCHL5 and NFRKB, and then affect the protein expression of NFRKB. Therefore, we first verified the binding effect of DRAIC and UCHL5 by RNA-IP assay, which is consistent with the predicted results of bioinformatics. At the same time, Co-IP assay confirmed the interaction between UCHL5 and NFRKB, which is in accord with the research by Nishi R [28]. On this basis, we observed the effect of DRAIC on the binding ability of UCHL5 and NFRKB, and found that DRAIC can significantly interfere with the binding ability of both. Naturally, NFRKB ubiquitination level has become the focus of attention. Through inhibiting the synthesis of total protein and the ubiquitin proteasome pathway, we found that DRAIC can significantly promote the degradation of NFRKB, and this effect will be blocked by proteasome inhibitors. Meanwhile, DRAIC can significantly promote the ubiquitin level of NFRKB. The biological effect is very important for translational medicine, so we investigated the effect of overexpression of DRAIC and recovery of NFRKB on the malignant proliferation and metastasis of GC. The results showed that DRAIC can significantly inhibit the proliferation and metastasis of GC cells, and this inhibitory effect can be antagonized by recovery of NFRKB.

## Conclusion

**Fig. 7:**
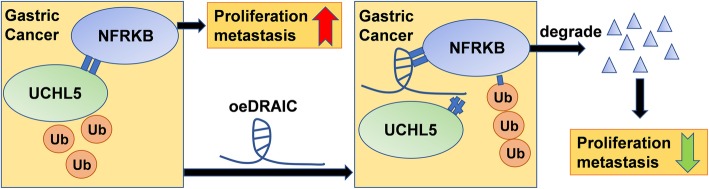
Schematic diagram of lncRNA DRAIC/UCHL5/NFRKB in GC

Combining the above results, it can be concluded that DRAIC mediates the ubiquitylation degradation of NFRKB by interfering with deubiquitination of NFRKB induced by UCHL5, and then exerts an anti-cancer effect in GC (Fig. 7). Our study revealed novel evidence for the interaction and biological effects of the lncRNA DRAIC/UCHL5/NFRKB pathway in GC, and provided new clues for the development of related diagnostic markers and targeted drugs.

## Supplementary information

**Additional file 1.**

## Data Availability

The datasets generated and analyzed during the current study are available in the [annolnc.cbi.pku.edu.cn/], [gepia.cancer-pku.cn/index.html] and [string-db.org/] repository. Partial data generated or analyzed during this study are included in this published article.

## Abbreviations

GC: Gastric cancer
lncRNA: Long non-coding RNA
DRAIC: Downregulated RNA in cancer
NFRKB: Nuclear factor related to kappaB binding protein
UCHL5: Ubiquitin C-terminal hydrolase L5
FBS: Fetal bovine serum
