# Supplementary figures and images for: LncRNA DRAIC inhibits proliferation and metastasis of gastric cancer cells through interfering with NFRKB deubiquitination mediated by UCHL5
Source: Cell Mol Biol Lett. 2020 Apr 25;25:29. doi: 10.1186/s11658-020-00221-0 (PMC7183705; doi:10.1186/s11658-020-00221-0)

Figure1

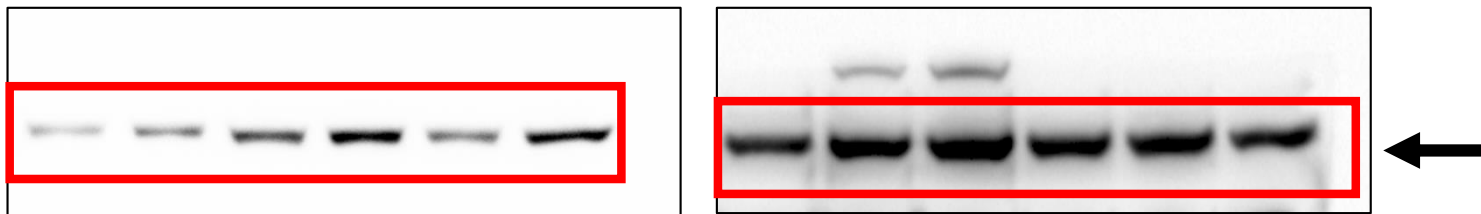

Figure2

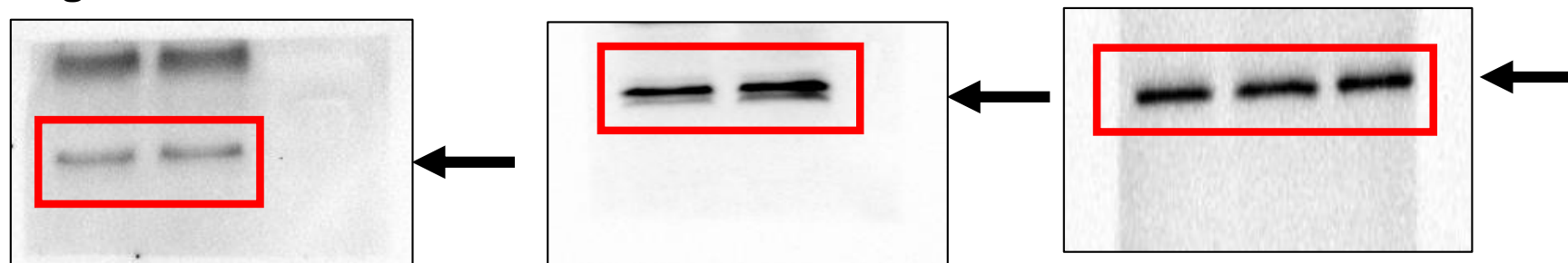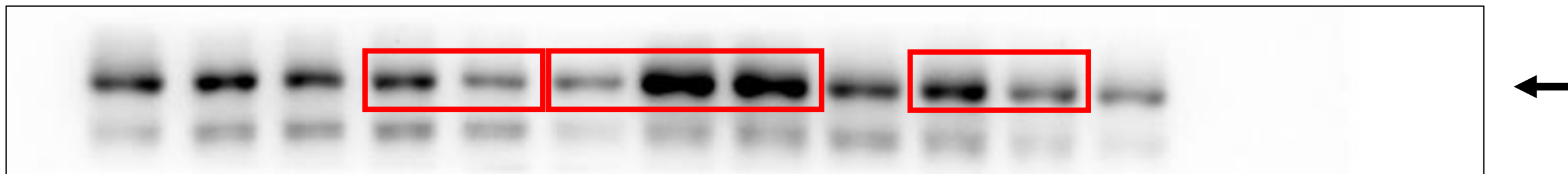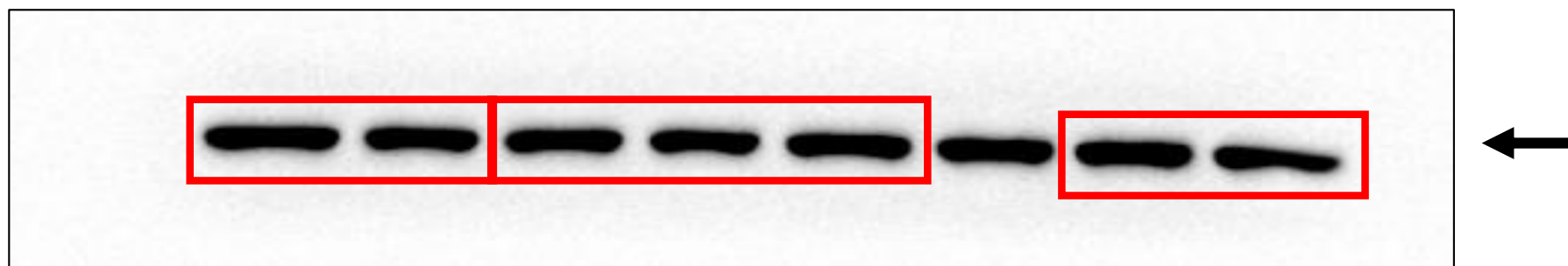

Figure3

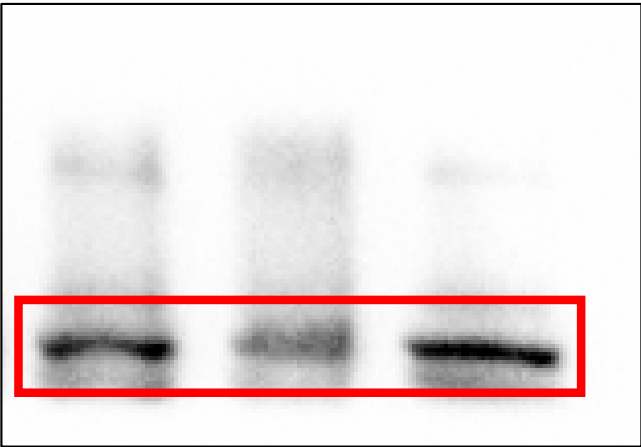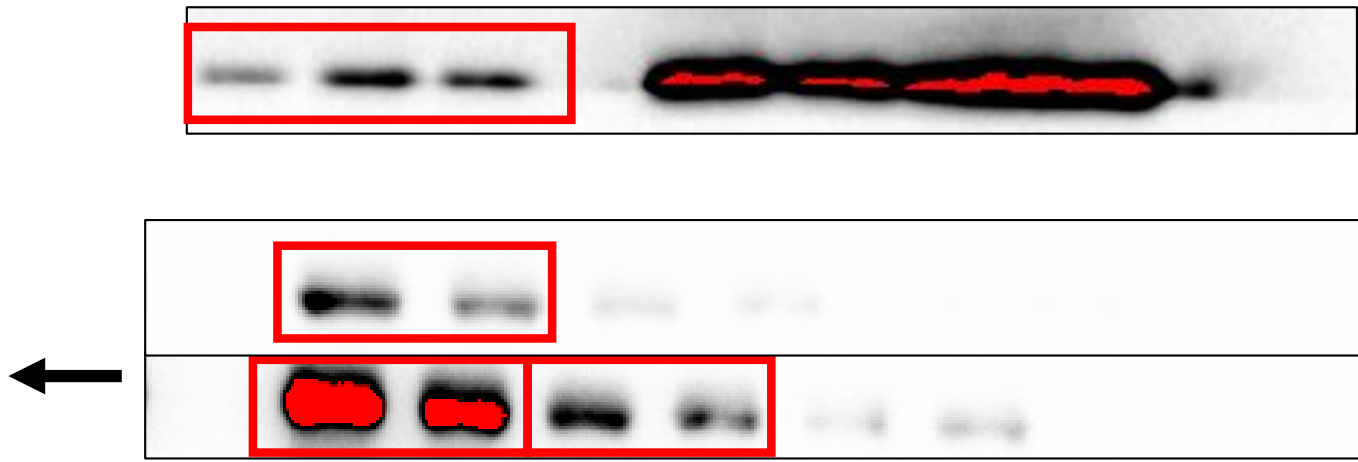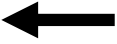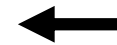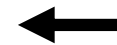

Low exposure

High exposure

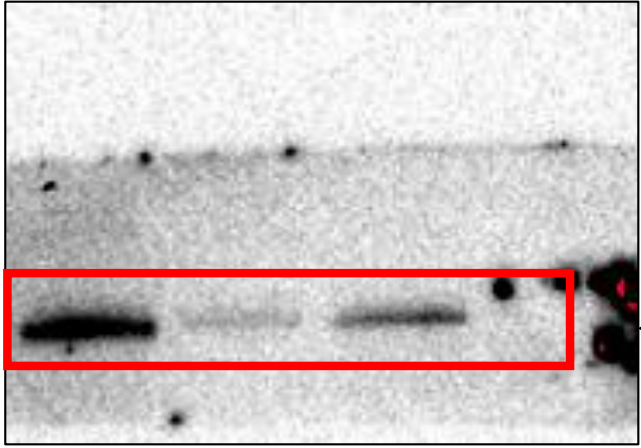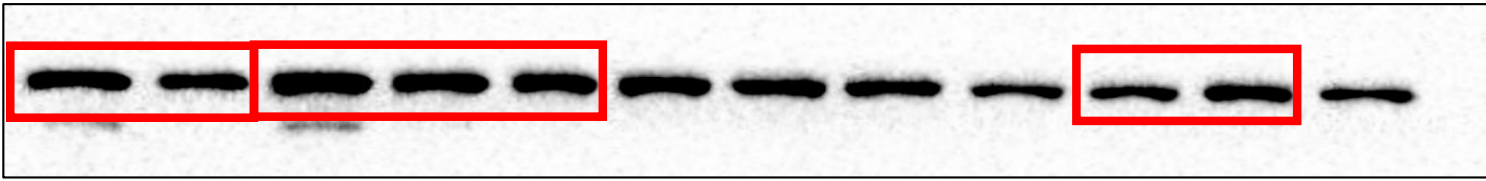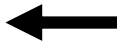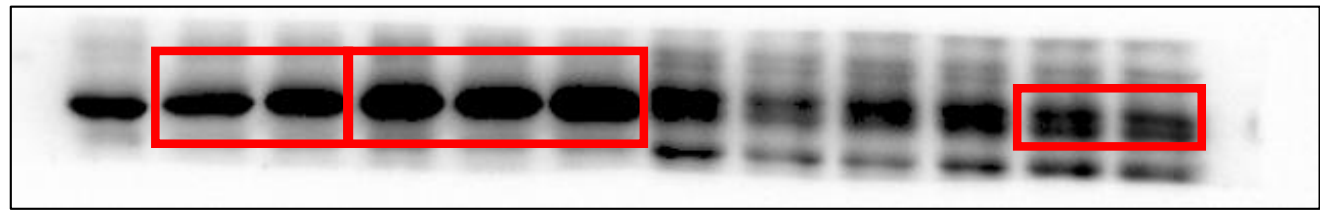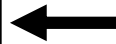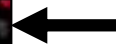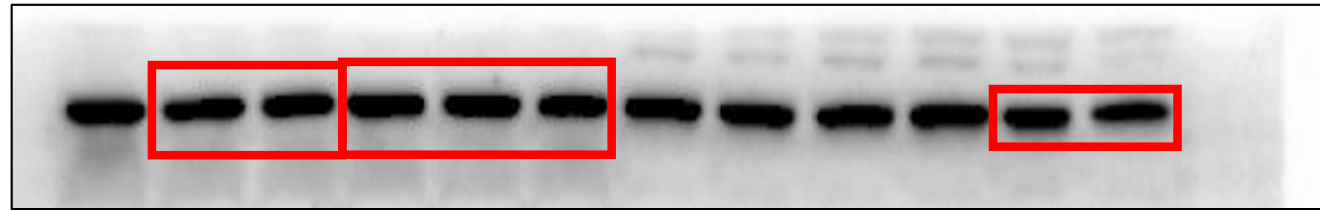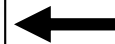

Figure4

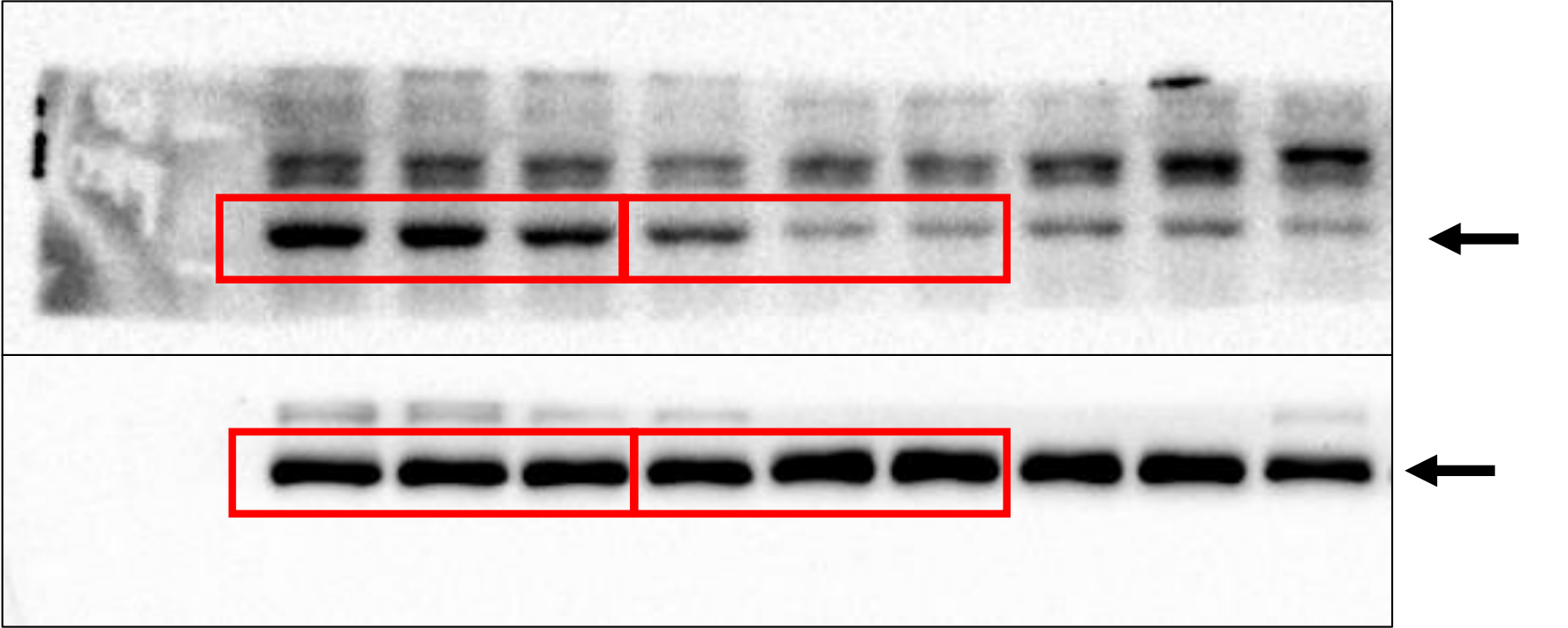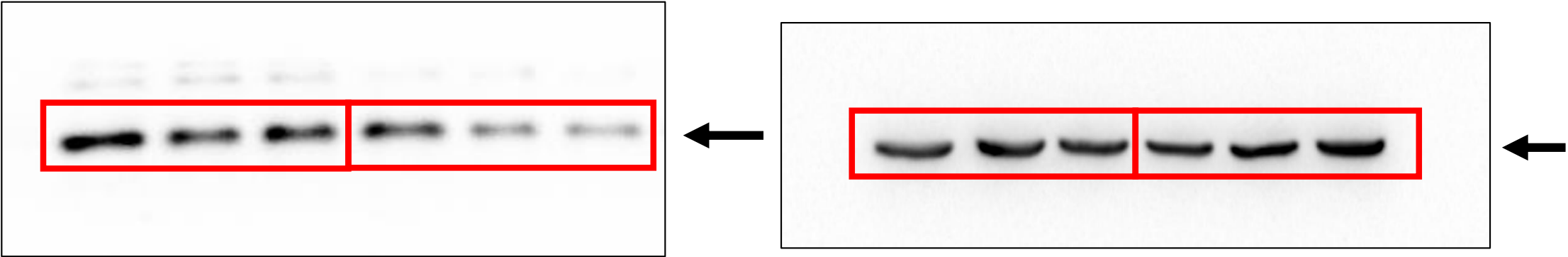

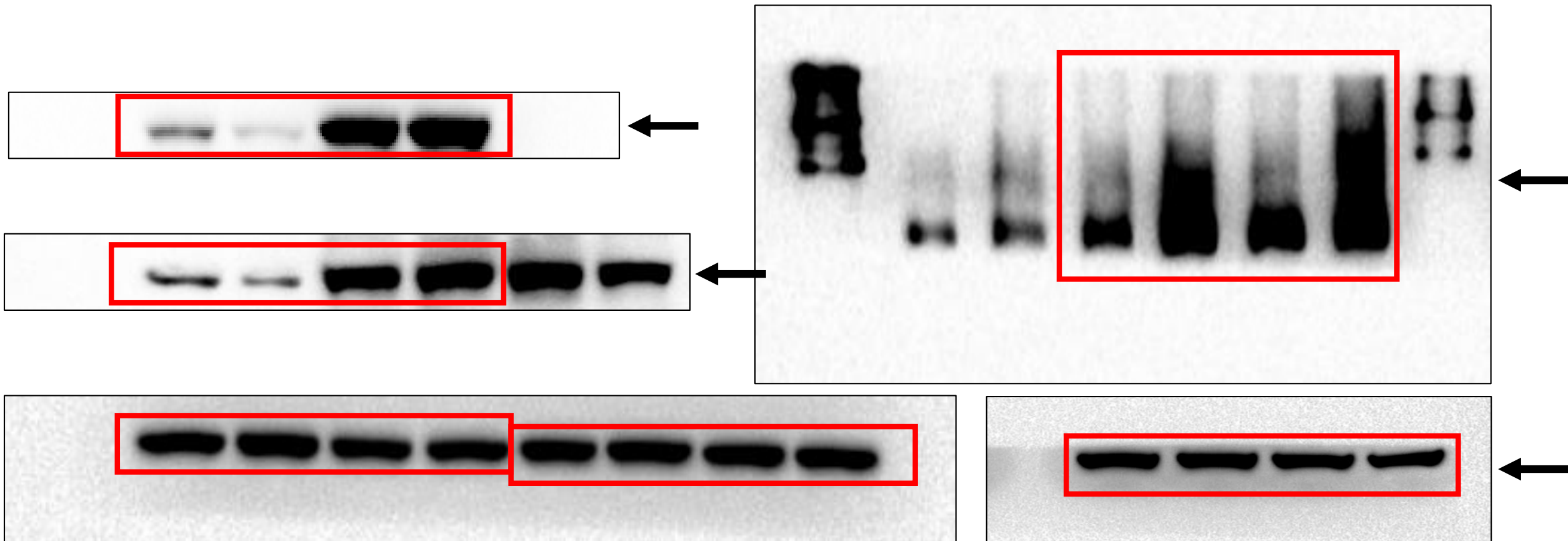

Supplement: Supplementary file 1 — Additional file 1. [file 11658_2020_221_MOESM1_ESM.pdf]
